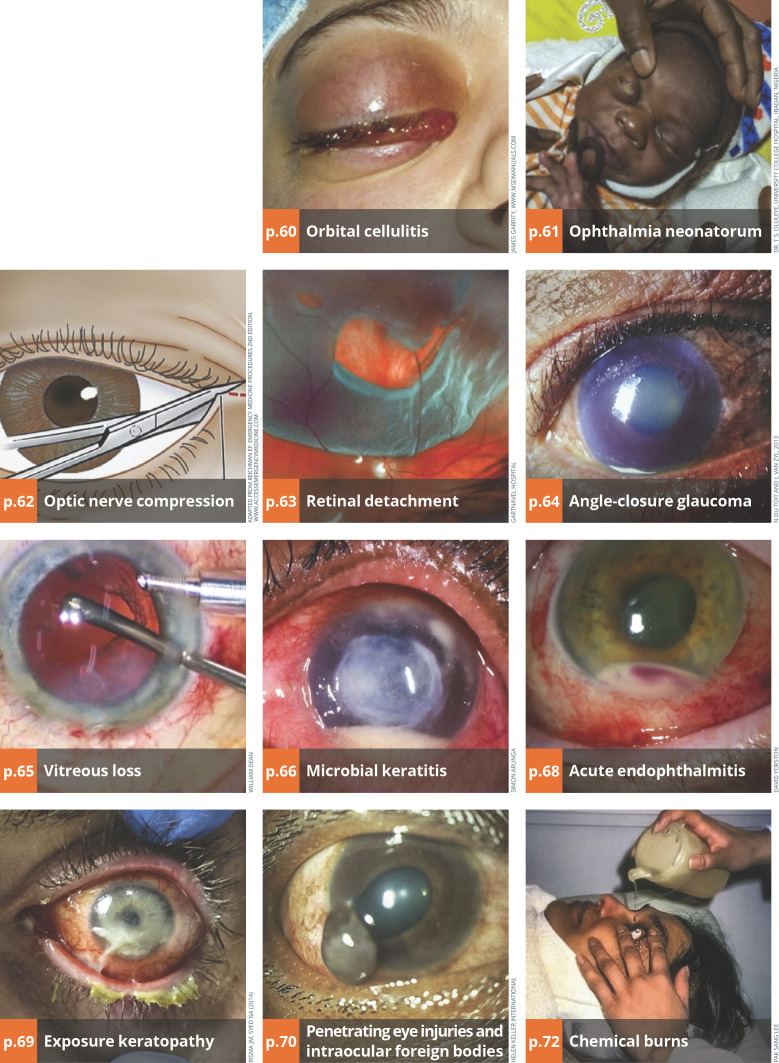# Eye emergencies in this issue

**Published:** 2018-11-09

**First-line management of and preparation for the following eye emergencies are addressed in this issue of the *Community Eye Health Journal*.**

**Figure:**